# Relationship between overweight and obesity and insufficient micronutrient intake: a nationwide study in Taiwan

**DOI:** 10.1017/jns.2023.31

**Published:** 2023-04-14

**Authors:** Shih-Pi Lin, Hsin-Yu Fang, Ming-Chieh Li

**Affiliations:** 1 Institute of Health Policy and Management, College of Public Health, National Taiwan University, Taipei, Taiwan; 2 Department of Public Health, College of Public Health, China Medical University, Taichung, Taiwan; 3 Department of Health Promotion and Health Education, College of Education, National Taiwan Normal University, Taipei, Taiwan

**Keywords:** Dietary reference intakes, Micronutrient deficiency, Nutrition and Health Survey in Taiwan, Obesity

## Abstract

The aim of the present study is to examine whether overweight or obese people in Taiwan have an inadequate intake of selected micronutrients. A population-based study was conducted using data from the Nutrition and Health Survey in Taiwan (NAHSIT) 2013–2016. We evaluated fourteen nutrient intakes using the 24 h dietary recall method. The dietary reference intake (DRI) adherence was estimated by the prevalence of participants whose intake was lower than the recommended dietary allowance (RDA) or adequate intakes (AIs) for selected micronutrients. Body mass index (BMI) ≥ 27 kg/m^2^ and waist circumference (WC), with men having WC ≥ 90 cm or women having WC ≥ 80 cm, were defined as obesity. A total of 3075 participants aged 19 years and above were included. After adjusting for confounders, we found that obese women have a lower DRI adherence of vitamin C (odds ratio (OR) 0⋅73, 95 % confidence interval (CI) 0⋅56, 0⋅95) and magnesium (OR 0⋅72, 95 % CI 0⋅54, 0⋅95), compared with normal-weight women. Obese men have a higher DRI adherence of vitamin B3 (OR 1⋅70, 95 % CI 1⋅29, 2⋅23), iron (OR 1⋅46, 95 % CI 1⋅06, 2⋅00) and zinc (OR 1⋅41, 95 % CI 1⋅07, 1⋅85), compared with normal-weight men. Similar findings were found using WC to define obesity. We conclude that obese women may have insufficient intakes of vitamin A, vitamin C and magnesium.

## Introduction

Obesity is a significant global public health concern, with the prevalence increasing from less than 3 % in 1975 to 11 % in 2016 among men and from 6 to 15 % among women^(1,2)^. It is associated with several chronic diseases, including cancer^(3)^, diabetes^(4,5)^, heart disease^(6)^ and respiratory diseases^(7)^. To address the consequences of the global obesity epidemic, it is crucial to understand how obesity leads to chronic diseases and develops effective prevention and treatment strategies.

The reasons behind obesity-related micronutrient deficiency remain unclear. Studies have suggested that overweight or obesity might affect micronutrient concentrations through imbalanced dietary intake, absorption, distribution, metabolism or excretion^(8–12)^. Overweight and obese adults have been found to have higher odds of low serum concentrations of micronutrients such as alpha-carotene, beta-carotene, beta-cryptoxanthin, lutein/zeaxanthin, total carotenoids, vitamin C, selenium and folate compared with normal-weight adults^(13)^. A study in Australia found that BMI was negatively correlated with serum concentrations of vitamin D, folate, magnesium and potassium^(14)^. Other studies have also reported varying levels of micronutrient deficiency among obese individuals^(15–19)^. Some studies have focused on micronutrient intake in overweight or obese individuals, with one finding no correlation between BMI and intake of micronutrients in both men and women^(14)^. Another study using the US National Health and Nutrition Examination Survey data found that vitamins A, B1, B2, B12 and D were negatively associated with the risk of obesity^(20)^.

Studies have indicated that obesity can cause imbalanced concentrations of micronutrients, potentially due to altered dietary intake, absorption, distribution, metabolism or excretion^(8–12)^. Prioritising dietary intake as the most modifiable factor from a public health perspective, we conducted a study using NAHSIT data to investigate the relationship between overweight/obesity and adherence to dietary reference intake (DRI) for selected micronutrients. Our hypothesis was that overweight/obese individuals might exhibit varied levels of adherence to DRI for these micronutrients.

## Methods

### Participants

The Nutrition and Health Survey in Taiwan (NAHSIT) was a population-representative survey in Taiwan investigating the nutritional status of the general population. We conducted a cross-sectional study using data from the NAHSIT 2013–2016. The survey method has been described elsewhere^(21)^. Briefly, a three-stage probability sampling strategy was used to achieve a representative sample for NAHSIT. The first stage was the selection of eight primary sampling units (townships and city districts) using probability proportional to size sampling methods. A total of 160 townships or city districts were chosen. The second stage was to randomly select several starting households to construct sampling clusters within each selected primary sampling unit. In the third stage, door-to-door visits were conducted to carry out interviews until the required number of the age and sex groups was reached. The Latin square design was used to allocate data collection times evenly over the four seasons to avoid seasonal variations that may affect dietary consumption and nutritional status.

The study population of NAHSIT was non-institutionalized Taiwanese nationals. NAHSIT also collected demographic data including age, sex, education, marital status and family income through face-to-face interviews. All participants attended a physical examination at a temporary health examination station, where body mass index (BMI) and waist circumference (WC) data were collected by either nurses or trained staff.

The diagram of the sample selection process is shown in Supplementary Figure S1. The number of participants in the NAHSIT survey was 11 072. The study participants in the present study were restricted to adults aged 19 and above according to the definition of adults adapted from the newest version of daily food guides (*n* 5770). Participants with complete demographic data, BMI and WC data, and 24 h dietary recall data were included in the final analysis, resulting in a total of 3075 study participants.

### Definition of overweight and obesity

Overweight or obesity was defined using BMI or WC according to the criteria of the Ministry of Health and Welfare in Taiwan^(22–24)^. Study participants were grouped into four groups: underweight (BMI < 18⋅5 kg/m^2^), normal (18⋅5 ≤ BMI < 24 kg/m^2^), overweight (24 ≤ BMI < 27 kg/m^2^) and obese (BMI ≥ 27 kg/m^2^). Moreover, we also used WC to define obesity and examine if similar results were observed. WC ≥ 90 cm for men and WC ≥ 80 cm for women were defined as obesity.

### Assessments of nutrient intakes

A validated 24 h dietary recall method was used to estimate the intakes of selected nutrients of study participants^(25–27)^. To address the potential intra-individual variance in estimating usual daily intakes using the 24 h recall data, repeated surveys were conducted for adjustment of the data. The NAHSIT randomly selected participants among different age groups (0–6, 7–12, 13–18, 19–64 and ≥65) from each sampling unit and conducted a repeated 24 h recall survey one week after the first survey. The correlations between two 24 h recall surveys were used to adjust the variance of daily dietary intakes^(27)^.

Fourteen nutrient intakes, including vitamins A, C, D, E, B1, B2, B3, B6 and B12, iron, magnesium, zinc, calcium and phosphorus were evaluated in NAHSIT. We did not evaluate the other nutrients was because that only these nutrient intake data were available in the NAHSIT dataset. In the present study, we examined the associations of overweight or obesity with the chance of adherence to the DRIs (8th edition in Taiwan). The DRIs were established by the Taiwan Food and Drug Administration (FDA) of the Ministry of Health and Welfare and were publicly accessible^(28)^. The DRIs for selected nutrients are shown in Supplementary Table S1. The DRI adherence was estimated by the prevalence of participants whose intake was lower than the recommended dietary allowance (RDA) or adequate intakes (AIs) for selected micronutrients.

### Statistical analysis

We categorised BMI into four groups (underweight, normal weight, overweight and obesity) based on standard definitions. We used the χ^2^ or Fisher's Exact test to examine the associations between different BMI categories and personal characteristics and conducted stratified analyses by sex. Confounding factors were selected based on prior knowledge and their potential influence on the exposures and outcomes. Finally, we fitted logistic regression models to assess the associations between BMI categories and inadequate intakes of micronutrients, adjusting for age (19 ≤ age ≤ 30, 31 ≤ age ≤ 50, 51 ≤ age ≤ 70 and age ≥ 71), education level (elementary school, junior high and high school, college or above), marital status (single, married or lived together, divorced, separated, widowed or refused to answer) and family income (income < NT $40 000, NT $40 000 ≤ income < NT $80 000, income ≥ NT $80 000, don't know or refuse to answer). All analyses were performed using SAS 9⋅4 (SAS Institute, Cary, NC).

## Results

A total of 3075 participants aged 19 and above were included in the final analysis (Table 1). Compared with normal-weight men, men with BMI of 27 kg/m^2^ and above were more likely to be older and to be married or lived together. Compared with normal-weight women, women with BMI of 27 kg/m^2^ and above were more likely to be older, tended to be less educated, more likely to be divorced, separated, widowed or refused to state marital status, and to have less family income. The median and interquartile range of micronutrient intakes among men and women are shown in Supplementary Table S2.

**Table 1.** Demographic descriptions of included study participants from the Nutrition and Health Survey in Taiwan (NAHSIT) 2013–2016 (*n* 3075)

Variables	Men					*P*-value
	Total	BMI < 18⋅5	18⋅5 ≤ BMI < 24	24 ≤ BMI < 27	BMI ≥ 27	
	*n* 1495	*n* 46	*n* 631	*n* 439	*n* 379	
Age						
19 ≤ age ≤ 30	196 (13⋅11)	15 (32⋅61)	104 (16⋅48)	37 (8⋅43)	40 (10⋅55)	<0⋅0001
31 ≤ age ≤ 50	354 (23⋅68)	12 (26⋅09)	145 (22⋅98)	93 (21⋅18)	104 (27⋅44)	
51 ≤ age ≤ 70	618 (41⋅34)	6 (13⋅04)	219 (34⋅71)	214 (48⋅75)	179 (47⋅23)	
Age ≥ 71	327 (21⋅87)	13 (28⋅26)	163 (25⋅83)	95 (21⋅64)	56 (14⋅78)	
Education						
Elementary school	805 (53⋅85)	25 (54⋅35)	316 (50⋅08)	256 (58⋅31)	208 (54⋅88)	0⋅07
Junior high and high school	341 (22⋅81)	8 (17⋅39)	148 (23⋅45)	90 (20⋅50)	95 (25⋅07)	
College or above	349 (23⋅34)	13 (28⋅26)	167 (26⋅47)	93 (21⋅19)	76 (20⋅05)	
Marital status						
Single	261 (17⋅46)	24 (52⋅17)	131 (20⋅76)	45 (10⋅25)	61 (16⋅09)	<0⋅0001
Married or lived together	1076 (71⋅97)	16 (34⋅78)	431 (68⋅30)	341 (77⋅68)	288 (75⋅99)	
Divorced, separated, widowed or refused to answer	158 (10⋅57)	6 (13⋅05)	69 (10⋅94)	53 (12⋅07)	30 (7⋅92)	
Family income						
Income < NT $40 000	452 (30⋅23)	14 (30⋅43)	194 (30⋅74)	136 (39⋅98)	108 (28⋅50)	0⋅52
NT $40 000 ≤ income < NT $80 000	381 (25⋅48)	14 (30⋅43)	166 (26⋅31)	105 (23⋅92)	96 (25⋅33)	
Income ≥ NT $80 000	350 (23⋅41)	6 (13⋅04)	147 (23⋅30)	110 (25⋅06)	87 (22⋅96)	
Don't know or refuse to answer	312 (20⋅88)	12 (26⋅10)	124 (19⋅65)	88 (20⋅04)	88 (23⋅21)	
Variables	Women					*P*-value
	Total	BMI < 18⋅5	18⋅5 ≤ BMI < 24	24 ≤ BMI < 27	BMI ≥ 27	
	*n* 1580	*n* 89	*n* 809	*n* 332	*n* 350	
Age						
19 ≤ age ≤ 30	249 (15⋅76)	38 (42⋅70)	151 (18⋅67)	22 (6⋅63)	38 (10⋅86)	<0⋅0001
31 ≤ age ≤ 50	410 (25⋅95)	25 (28⋅09)	247 (30⋅53)	70 (21⋅08)	68 (19⋅43)	
51 ≤ age ≤ 70	642 (40⋅63)	15 (16⋅85)	293 (36⋅22)	168 (50⋅60)	166 (47⋅43)	
Age ≥ 71	279 (17⋅66)	11 (12⋅36)	118 (14⋅59)	72 (21⋅69)	78 (22⋅29)	
Education						
Elementary school	734 (46⋅46)	47 (52⋅81)	383 (47⋅34)	162 (48⋅80)	142 (40⋅57)	<0⋅0001
Junior high and high school	535 (33⋅86)	15 (16⋅85)	214 (26⋅45)	132 (39⋅76)	174 (49⋅71)	
College or above	311 (19⋅68)	27 (30⋅34)	212 (26⋅21)	38 (11⋅45)	34 (9⋅71)	
Marital status						
Single	255 (16⋅14)	34 (38⋅20)	153 (18⋅91)	28 (8⋅43)	40 (11⋅43)	<0⋅0001
Married or lived together	1008 (63⋅80)	43 (48⋅31)	518 (64⋅03)	233 (70⋅18)	214 (61⋅14)	
Divorced, separated, widowed or refused to answer	317 (20⋅06)	12 (13⋅48)	138 (17⋅06)	71 (21⋅39)	96 (27⋅43)	
Family income						
Income < NT $40 000	457 (28⋅92)	25 (28⋅09)	204 (25⋅22)	97 (29⋅22)	131 (37⋅43)	<0⋅01
NT $40 000 ≤ income < NT $80 000	362 (22⋅91)	27 (30⋅34)	183 (22⋅62)	84 (25⋅30)	68 (19⋅43)	
Income ≥ NT $80 000	355 (22⋅47)	22 (24⋅72)	220 (27⋅19)	68 (20⋅48)	45 (12⋅86)	
Don't know or refuse to answer	406 (25⋅70)	15 (16⋅85)	202 (24⋅97)	83 (25⋅00)	106 (30⋅28)	

NT, the New Taiwan dollars; BMI, body mass index.

The associations of baseline personal characteristics with different BMI groups were evaluated using χ^2^ or Fisher's Exact tests.

After adjusting for age, education level, marital status and family income, we found that obese men have a higher DRI adherence for vitamin B3 (odds ratio (OR) 1⋅70, 95 % confidence interval (CI) 1⋅29, 2⋅23; *P*-value < 0⋅001), Fe (OR 1⋅46, 95 % CI 1⋅06, 2⋅00; *P*-value = 0⋅02) and Zn (OR 1⋅41, 95 % CI 1⋅07, 1⋅85; *P*-value = 0⋅01), compared with normal-weight men. However, compared with normal-weight women, obese women have lower DRI adherences for vitamin C (OR 0⋅73, 95 % CI 0⋅56, 0⋅95; *P*-value = 0⋅02) and Mg (OR 0⋅72, 95 % CI 0⋅54, 0⋅95; *P*-value = 0⋅02). For the most micronutrient intakes, we found no difference between obese and normal-weight people in DRI adherence (Table 2).

**Table 2.** The associations between BMI groups and DRI adherence of selected micronutrients using data from the Nutrition and Health Survey in Taiwan (NAHSIT) 2013–2016

Nutrient intakes	Men (*n* 1495)				
	BMI < 18⋅5	18⋅5 ≤ BMI < 24	24 ≤ BMI < 27	BMI ≥ 27	BMI ≥ 24
Vitamin A	0⋅62 (0⋅33–1⋅15)	1	0⋅83 (0⋅65–1⋅06)	0⋅88 (0⋅68–1⋅14)	0⋅85 (0⋅69–1⋅05)
Vitamin C	0⋅95 (0⋅51–1⋅76)	1	1⋅00 (0⋅78–1⋅29)	0⋅97 (0⋅74–1⋅26)	0⋅98 (0⋅79–1⋅22)
Vitamin D	0⋅54 (0⋅19–1⋅57)	1	0⋅97 (0⋅69–1⋅37)	0⋅77 (0⋅53–1⋅12)	0⋅88 (0⋅65–1⋅18)
Vitamin E	0⋅68 (0⋅26–1⋅80)	1	0⋅96 (0⋅68–1⋅34)	0⋅96 (0⋅67–1⋅38)	0⋅96 (0⋅72–1⋅28)
Vitamin B1	0⋅86 (0⋅46–1⋅59)	1	1⋅11 (0⋅86–1⋅43)	1⋅22 (0⋅93–1⋅59)	1⋅16 (0⋅93–1⋅44)
Vitamin B2	0⋅86 (0⋅46–1⋅61)	1	0⋅92 (0⋅71–1⋅19)	1⋅00 (0⋅77–1⋅30)	0⋅96 (0⋅77–1⋅19)
Vitamin B6	1⋅20 (0⋅62–2⋅33)	1	1⋅11 (0⋅84–1⋅45)	1⋅10 (0⋅83–1⋅45)	1⋅10 (0⋅88–1⋅39)
Vitamin B12	1⋅05 (0⋅55–2⋅00)	1	0⋅96 (0⋅74–1⋅24)	1⋅21 (0⋅92–1⋅59)	1⋅06 (0⋅85–1⋅33)
Vitamin B3	1⋅41 (0⋅74–2⋅66)	1	1⋅27 (0⋅98–1⋅64)	**1⋅70** (**1⋅29–2⋅23)**	**1⋅45** (**1⋅16–1⋅80)**
Iron	0⋅81 (0⋅42–1⋅56)	1	**1⋅41** (**1⋅04–1⋅90)**	**1⋅46** (**1⋅06–2⋅00)**	**1⋅43** (**1⋅11–1⋅84)**
Magnesium	0⋅65 (0⋅32–1⋅33)	1	1⋅11 (0⋅86–1⋅44)	1⋅12 (0⋅85–1⋅47)	1⋅11 (0⋅89–1⋅39)
Zinc	1⋅28 (0⋅67–2⋅43)	1	1⋅09 (0⋅83–1⋅42)	**1⋅41** (**1⋅07–1⋅85)**	1⋅23 (0⋅98–1⋅54)
Calcium	0⋅72 (0⋅27–1⋅90)	1	0⋅89 (0⋅61–1⋅28)	1⋅02 (0⋅69–1⋅50)	0⋅94 (0⋅69–1⋅29)
Phosphorus	0⋅98 (0⋅48–2⋅02)	1	1⋅26 (0⋅92–1⋅73)	1⋅32 (0⋅94–1⋅83)	1⋅29 (0⋅99–1⋅68)

Nutrient intakes	Women (*n* 1580)				
	BMI < 18⋅5	18⋅5 ≤ BMI < 24	24 ≤ BMI < 27	BMI ≥ 27	BMI ≥ 24
Vitamin A	1⋅11 (0⋅70–1⋅75)	1	**0⋅65** (**0⋅50–0⋅85)**	0⋅86 (0⋅66–1⋅12)	**0⋅75** (**0⋅61–0⋅93)**
Vitamin C	**0⋅59** (**0⋅37–0⋅93)**	1	0⋅91 (0⋅70–1⋅19)	**0⋅73** (**0⋅56–0⋅95)**	0⋅81 (0⋅65–1⋅01)
Vitamin D	0⋅85 (0⋅41–1⋅76)	1	1⋅09 (0⋅72–1⋅66)	0⋅99 (0⋅64–1⋅52)	1⋅04 (0⋅74–1⋅46)
Vitamin E	1⋅01 (0⋅50–2⋅05)	1	1⋅16 (0⋅79–1⋅70)	0⋅93 (0⋅62–1⋅40)	1⋅05 (0⋅76–1⋅44)
Vitamin B1	0⋅86 (0⋅54–1⋅37)	1	0⋅90 (0⋅69–1⋅18)	1⋅04 (0⋅79–1⋅36)	0⋅97 (0⋅78–1⋅20)
Vitamin B2	**0⋅62** (**0⋅39–0⋅98)**	1	0⋅89 (0⋅68–1⋅16)	0⋅93 (0⋅72–1⋅21)	0⋅91 (0⋅74–1⋅13)
Vitamin B3	0⋅81 (0⋅52–1⋅27)	1	0⋅91 (0⋅70–1⋅19)	1⋅00 (0⋅77–1⋅30)	0⋅95 (0⋅77–1⋅18)
Vitamin B6	**0⋅62** (**0⋅39–0⋅97)**	1	0⋅96 (0⋅73–1⋅25)	0⋅80 (0⋅62–1⋅04)	0⋅87 (0⋅71–1⋅08)
Vitamin B12	0⋅67 (0⋅43–1⋅06)	1	0⋅99 (0⋅76–1⋅29)	1⋅14 (0⋅88–1⋅49)	1⋅06 (0⋅86–1⋅32)
Iron	0⋅95 (0⋅59–1⋅52)	1	0⋅89 (0⋅68–1⋅17)	1⋅01 (0⋅77–1⋅33)	0⋅95 (0⋅76–1⋅18)
Magnesium	0⋅87 (0⋅53–1⋅43)	1	0⋅98 (0⋅75–1⋅29)	**0⋅72** (**0⋅54–0⋅95)**	0⋅84 (0⋅67–1⋅06)
Zinc	**0⋅53** (**0⋅30–0⋅95)**	1	1⋅23 (0⋅92–1⋅65)	1⋅02 (0⋅76–1⋅38)	1⋅12 (0⋅88–1⋅43)
Calcium	0⋅97 (0⋅42–2⋅19)	1	0⋅94 (0⋅60–1⋅47)	0⋅80 (0⋅50–1⋅30)	0⋅87 (0⋅60–1⋅26)
Phosphorus	0⋅71 (0⋅44–1⋅14)	1	0⋅82 (0⋅62–1⋅08)	0⋅96 (0⋅73–1⋅27)	0⋅89 (0⋅71–1⋅11)

BMI, body mass index. Exposure variable: BMI groups; Outcome variable: DRI adherence. All the logistic regression models were adjusted for age, education level, marital status and family income. The values were presented as odds ratio (95 % confidence interval).

Bold values are statistically significant.

Table 3 shows similar findings using WC to define obesity. After adjusting for potential confounding factors, men with WC ≥ 90 cm had higher DRI adherences of vitamin B3 (OR 1⋅22, 95 % CI 1⋅01, 1⋅47; *P*-value = 0⋅04), vitamin B12 (OR 1⋅29, 95 % CI 1⋅07, 1⋅57; *P*-value < 0⋅01) and Fe (OR 1⋅33, 95 % CI 1⋅07, 1⋅65; *P*-value = 0⋅01). In contrast, women with WC ≥ 80 cm had lower DRI adherences of vitamin A (OR 0⋅82, 95 % CI 0⋅68, 1⋅00; *P*-value = 0⋅05) and Mg (OR 0⋅78, 95 % CI 0⋅64, 0⋅97; *P*-value = 0⋅02). For the other micronutrient intakes, we found no difference between obese and non-obese people in DRI adherence.

**Table 3.** The associations between WC groups and DRI adherence of selected micronutrients using data from the Nutrition and Health Survey in Taiwan (NAHSIT) 2013–2016

Nutrient intakes	Men (*n* 1495)	
	WC < 90	WC ≥ 90
Vitamin A	1	0⋅92 (0⋅76–1⋅10)
Vitamin C	1	0⋅93 (0⋅77–1⋅12)
Vitamin D	1	0⋅89 (0⋅69–1⋅17)
Vitamin E	1	0⋅97 (0⋅75–1⋅26)
Vitamin B1	1	1⋅16 (0⋅96–1⋅40)
Vitamin B2	1	1⋅01 (0⋅84–1⋅23)
Vitamin B6	1	1⋅08 (0⋅89–1⋅31)
Vitamin B12	1	**1⋅29** (**1⋅07–1⋅57)**
Vitamin B3	1	**1⋅22** (**1⋅01–1⋅47)**
Iron	1	**1⋅33** (**1⋅07–1⋅65)**
Magnesium	1	1⋅04 (0⋅85–1⋅26)
Zinc	1	1⋅05 (0⋅86–1⋅28)
Calcium	1	0⋅82 (0⋅61–1⋅09)
Phosphorus	1	1⋅12 (0⋅90–1⋅40)

Nutrient intakes	Women (*n* 1580)	
	WC < 80	WC ≥ 80
Vitamin A	1	**0⋅82** (**0⋅68–1⋅00)**
Vitamin C	1	0⋅84 (0⋅69–1⋅02)
Vitamin D	1	0⋅86 (0⋅63–1⋅19)
Vitamin E	1	0⋅77 (0⋅57–1⋅05)
Vitamin B1	1	0⋅93 (0⋅76–1⋅13)
Vitamin B2	1	1⋅02 (0⋅84–1⋅24)
Vitamin B3	1	0⋅95 (0⋅78–1⋅15)
Vitamin B6	1	0⋅89 (0⋅73–1⋅07)
Vitamin B12	1	0⋅98 (0⋅81–1⋅19)
Iron	1	0⋅93 (0⋅76–1⋅14)
Magnesium	1	**0⋅78** (**0⋅64–0⋅97)**
Zinc	1	0⋅97 (0⋅78–1⋅21)
Calcium	1	0⋅76 (0⋅54–1⋅08)
Phosphorus	1	0⋅83 (0⋅68–1⋅02)

WC, waist circumference. Exposure variable: BMI groups; Outcome variable: DRI adherence. All the logistic regression models were adjusted for age, education level, marital status and family income. The values were presented as odds ratio (95 % confidence interval).

Bold values are statistically significant.

## Discussion

In the present study, we found that men who were obese or overweight had a higher chance of adhering to the DRIs for vitamin B3, vitamin B12, iron and zinc, but not for most of the other selected nutrients. In contrast, obese women had a higher chance of having insufficient intakes of vitamin A, vitamin C and magnesium compared with normal-weight women. Similar findings were observed using WC as an indicator of obesity. One possible explanation is that obese individuals are more likely to consume high-energy foods that are usually nutritionally poor^(29)^. The most important contribution of our study is that we did not merely examine the associations between micronutrient intakes and obesity. Instead, we examined the associations between the chance of adherence to the official DRIs and obesity and found insufficient intakes of some micronutrients among obese women. We argue that this approach is superior because a simple negative association between specific micronutrient intakes and obesity cannot determine whether a deficiency exists.

Overweight and obesity have been linked to micronutrient deficiencies in previous studies, which typically proposed possible explanations from a biological mechanism standpoint^(30)^. For instance, research has found that iron and vitamin D are sequestered in adipose tissues in obese individuals because iron is redistributed to different tissues^(30–32)^. Obesity may also lead to adipocyte dysfunction, which impairs adipose tissue functionality, sequestering fat-soluble vitamins in the adipocyte and reducing micronutrient concentrations in blood^(30,33)^. Many studies have focused on serum concentrations of micronutrients and their associations with obesity. In China, a study found that obese individuals had lower serum concentrations of vitamin A^(34)^. García *et al.* reported that serum concentrations of vitamin C were negatively associated with BMI and waist-to-height ratio^(35)^. Several studies have also found that serum magnesium was lower in obese or overweight children^(36–39)^, adolescents^(40)^ and women^(41)^. Other studies have shown varying levels of micronutrient deficiency among obese individuals concerning different micronutrients^(14–19)^. However, relatively few studies have examined micronutrient intakes among overweight or obese individuals. For example, one study assessed correlations between BMI and dietary intakes of micronutrients and found no linear correlation between BMI and intake of micronutrients among both men and women^(14)^, which contrasts with our findings. Another study found that obese individuals may develop hypoferremia due to inadequate iron intake relative to their body weight^(31)^, which contradicts our findings in men, where we found that obese men had a higher chance of adhering to the DRIs for iron. A recent study that used data from the US National Health and Nutrition Examination Survey found that vitamins A, B1, B2, B12 and D were negatively associated with the risk of obesity. Furthermore, when the concentrations of all nine vitamins (A, B1, B2, B6, B12, C, D, E and K) were at or above the 55th percentile, the combined intake of them might reduce the risk of obesity in both children and adolescents compared with the median values^(20)^. Studies on the associations between magnesium intake and obesity have been inconsistent. A cohort study of American young adults found a negative correlation between magnesium intake and obesity incidence^(42)^. Another study using data from the National Health and Nutrition Examination Survey also found that magnesium intake was negatively correlated with BMI and WC^(43)^. In contrast, another study found no association in magnesium intake between obese women and healthy controls^(44)^, which contrasts with our findings. However, as we have mentioned, although previous studies have found negative associations between certain micronutrient intake and obesity, it does not necessarily mean that micronutrient deficiency is more prevalent among obese individuals. Our study provides further evidence that obese women have insufficient intakes of vitamins A, C and magnesium according to the official DRIs.

Our findings indicate that obese men and women exhibit different patterns of DRI adherence, although the underlying reason is not clear. It is possible that men and women have distinct dietary choices, as suggested by previous studies^(45,46)^. For instance, one study found that women were more likely to consume legumes, vegetables, fruits, eggs, milk, vegetable oils and added sugar. In contrast, men may consume more starchy foods, soft drinks and alcoholic beverages^(45)^. Our study further demonstrates that obesity status may also impact the dietary choices of men and women, resulting in varying patterns of micronutrient intake. Thus, when conducting dietary intervention trials, obesity status should be considered.

Our study has both strengths and limitations. The strengths of the study include a fair sample size to assess the potential association between obesity and the chance of adherence to the DRIs. Furthermore, the population-based design allows for generalisation of our findings to the public. However, the study also has limitations. Firstly, the cross-sectional design does not allow for establishing causality. Secondly, we only had a one-time dietary assessment, which may introduce misclassification of nutrient intakes. Non-differential misclassification might favour the null hypothesis. However, the NAHSIT performed repeated surveys to adjust the 24 h recall data and minimise potential bias. Thirdly, we did not examine the associations of blood or urinary biomarkers of nutrients with obesity status. One reason for this is that the concentrations of nutrient biomarkers are affected not only by the individual's intake of nutrients but also by their absorption, distribution, metabolism or excretion of micronutrients. An association found between concentrations of certain nutrient biomarkers and obesity might not allow for any conclusion due to the complicated mechanisms. Additionally, many studies have already examined the serum concentrations of nutrient biomarkers among obese individuals, while studies on the role of dietary intake remain limited. We believe that our study contributes to filling this knowledge gap.

In conclusion, our study found that Taiwanese women with overweight or obesity may experience insufficient intake of some micronutrients. Further prospective studies are needed to confirm our findings. Moreover, future research could examine the intakes of both micronutrients and nutrient biomarkers simultaneously among overweight or obese individuals.

## Supplementary material

For supplementary material accompanying this paper visit https://doi.org/10.1017/jns.2023.31. click here to view supplementary material

## References

[1] NCD Risk Factor Collaboration (NCD-RisC) (2017) Worldwide trends in body-mass index, underweight, overweight, and obesity from 1975 to 2016: a pooled analysis of 2416 population-based measurement studies in 128⋅9 million children, adolescents, and adults. Lancet 390, 2627–2642. 29029897 10.1016/S0140-6736(17)32129-3 PMC5735219

[2] Jaacks LM, Vandevijvere S, Pan A, (2019) The obesity transition: stages of the global epidemic. Lancet Diabetes Endocrinol 7, 231–240. 30704950 10.1016/S2213-8587(19)30026-9 PMC7360432

[3] Calle EE & Kaaks R (2004) Overweight, obesity and cancer: epidemiological evidence and proposed mechanisms. Nat Rev Cancer 4, 579–591. 15286738 10.1038/nrc1408

[4] Verma S & Hussain ME (2017) Obesity and diabetes: an update. Diabetes Metab Syndr 11, 73–79. 27353549 10.1016/j.dsx.2016.06.017

[5] Hossain P, Kawar B & El Nahas M (2007) Obesity and diabetes in the developing world—a growing challenge. N Engl J Med 356, 213–215. 17229948 10.1056/NEJMp068177

[6] Lavie CJ, Arena R, Alpert MA, (2018) Management of cardiovascular diseases in patients with obesity. Nat Rev Cardiol 15, 45–56. 28748957 10.1038/nrcardio.2017.108

[7] Peters U, Suratt BT, Bates JH, (2018) Beyond BMI: obesity and lung disease. Chest 153, 702–709. 28728934 10.1016/j.chest.2017.07.010 PMC5989645

[8] Olusi SO (2002) Obesity is an independent risk factor for plasma lipid peroxidation and depletion of erythrocyte cytoprotectic enzymes in humans. Int J Obes Relat Metab Disord 26, 1159–1164. 12187391 10.1038/sj.ijo.0802066

[9] Patrini C, Griziotti A & Ricciardi L (2004) Obese individuals as thiamin storers. Int J Obes Relat Metab Disord 28, 920–924. 15098017 10.1038/sj.ijo.0802638

[10] Johnston CS (2005) Strategies for healthy weight loss: from vitamin C to the glycemic response. J Am Coll Nutr 24, 158–165. 15930480 10.1080/07315724.2005.10719460

[11] Snijder MB, van Dam RM, Visser M, (2005) Adiposity in relation to vitamin D status and parathyroid hormone levels: a population-based study in older men and women. J Clin Endocrinol Metab 90, 4119–4123. 15855256 10.1210/jc.2005-0216

[12] Ledikwe JH, Blanck HM, Khan LK, (2006) Low-energy-density diets are associated with high diet quality in adults in the United States. J Am Diet Assoc 106, 1172–1180. 16863711 10.1016/j.jada.2006.05.013

[13] Kimmons JE, Blanck HM, Tohill BC, (2006) Associations between body mass index and the prevalence of low micronutrient levels among US adults. Med Gen Med 8, 59. PMC1868363 17415336

[14] McKay J, Ho S, Jane M, (2020) Overweight & obese Australian adults and micronutrient deficiency. BMC Nutr 6, 1–13. 32377370 10.1186/s40795-020-00336-9 PMC7193396

[15] Moor de Burgos A, Wartanowicz M & Ziemlański S (1992) Blood vitamin and lipid levels in overweight and obese women. Eur J Clin Nutr 46, 803–808. 1425534

[16] Hötzel D (1986) Suboptimal nutritional status in obesity (selected nutrients). Bibl Nutr Diet 37, 36–41. 3942587

[17] Reitman A, Friedrich I, Ben-Amotz A, (2002) Low plasma antioxidants and normal plasma B vitamins and homocysteine in patients with severe obesity. Isr Med Assoc J 4, 590–593. 12183861

[18] Wallström P, Wirfält E, Lahmann PH, (2001) Serum concentrations of beta-carotene and alpha-tocopherol are associated with diet, smoking, and general and central adiposity. Am J Clin Nutr 73, 777–785. 11273853 10.1093/ajcn/73.4.777

[19] Al-Delaimy WK, van Kappel AL, Ferrari P, (2004) Plasma levels of six carotenoids in nine European countries: report from the European Prospective Investigation into Cancer and Nutrition (EPIC). Public Health Nutr 7, 713–722. 15369608 10.1079/phn2004598

[20] Tang W, Zhan W, Wei M, (2021) Associations between different dietary vitamins and the risk of obesity in children and adolescents: a machine learning approach. Front Endocrinol (Lausanne) 12, 816975. 35250848 10.3389/fendo.2021.816975 PMC8893992

[21] Li MC & Fang HY (2020) Adherence to daily food guides is associated with lower risk of metabolic syndrome: the Nutrition and Health Survey in Taiwan. Nutrients 12, e2955. 10.3390/nu12102955 PMC7600265 32992533

[22] Li WC, Chen IC, Chang YC, (2013) Waist-to-height ratio, waist circumference, and body mass index as indices of cardiometabolic risk among 36,642 Taiwanese adults. Eur J Nutr 52, 57–65. 22160169 10.1007/s00394-011-0286-0 PMC3549404

[23] Chu NF (2005) Prevalence of obesity in Taiwan. Obes Rev 6, 271–274. 16246212 10.1111/j.1467-789X.2005.00175.x

[24] Lin W, Lee L, Chen C, (2002) Optimal cut-off values for obesity: using simple anthropometric indices to predict cardiovascular risk factors in Taiwan. Int J Obes 26, 1232–1238. 10.1038/sj.ijo.0802040 12187401

[25] Huang YC, Lee MS, Pan WH, (2011) Validation of a simplified food frequency questionnaire as used in the Nutrition and Health Survey in Taiwan (NAHSIT) for the elderly. Asia Pac J Clin Nutr 20, 134–140. 21393121

[26] Pan W-H, Chang YH, Chen JY, (1999) Nutrition and Health Survey in Taiwan (NAHSIT) 1993–1996: dietary nutrient intakes assessed by 24-hour recall. Nutr Sci J 24, 11–39.

[27] Chang HY, Suchindran CM & Pan WH (2001) Using the overdispersed exponential family to estimate the distribution of usual daily intakes of people aged between 18 and 28 in Taiwan. Stat Med 20, 2337–2350. 11468767 10.1002/sim.838

[28] Taiwan HPA (2020) Dietary Reference Intakes Eighth Edition. https://www.hpa.gov.tw/Pages/Detail.aspx?nodeid=4248&pid=12285

[29] Ernst B, Thurnheer M, Schmid SM, (2009) Evidence for the necessity to systematically assess micronutrient status prior to bariatric surgery. Obes Surg 19, 66–73. 18491197 10.1007/s11695-008-9545-4

[30] García OP, Long KZ & Rosado JL (2009) Impact of micronutrient deficiencies on obesity. Nutr Rev 67, 559–572. 19785688 10.1111/j.1753-4887.2009.00228.x

[31] Yanoff LB, Menzie CM, Denkinger B, (2007) Inflammation and iron deficiency in the hypoferremia of obesity. Int J Obes (Lond) 31, 1412–1419. 17438557 10.1038/sj.ijo.0803625 PMC2266872

[32] Wortsman J, Matsuoka LY, Chen TC, (2000) Decreased bioavailability of vitamin D in obesity. Am J Clin Nutr 72, 690–693. 10966885 10.1093/ajcn/72.3.690

[33] Haugen F & Drevon CA (2007) The interplay between nutrients and the adipose tissue. Proc Nutr Soc 66, 171–182. 17466100 10.1017/S0029665107005423

[34] Wei X, Peng R, Cao J, (2016) Serum vitamin A status is associated with obesity and the metabolic syndrome among school-age children in Chongqing, China. Asia Pac J Clin Nutr 25, 563–570. 27440692 10.6133/apjcn.092015.03

[35] García OP, Ronquillo D, Caamaño Mdel C, (2012) Zinc, vitamin A, and vitamin C status are associated with leptin concentrations and obesity in Mexican women: results from a cross-sectional study. Nutr Metab (Lond) 9, 59. 22703731 10.1186/1743-7075-9-59 PMC3406981

[36] Hassan SAU, Ahmed I, Nasrullah A, (2017) Comparison of serum magnesium levels in overweight and obese children and normal weight children. Cureus 9, e1607. 29075585 10.7759/cureus.1607 PMC5654973

[37] Jose B, Jain V, Vikram NK, (2012) Serum magnesium in overweight children. Indian Pediatr 49, 109–112. 21719932 10.1007/s13312-012-0024-6

[38] Zaakouk AM, Hassan MA & Tolba OA (2016) Serum magnesium status among obese children and adolescents. Egypt Pediatr Assoc Gaz 64, 32–37.

[39] Celik N, Andiran N & Yilmaz AE (2011) The relationship between serum magnesium levels with childhood obesity and insulin resistance: a review of the literature. J Pediatr Endocrinol Metab 24, 675–678. 22145455

[40] Suliburska J, Cofta S, Gajewska E, (2013) The evaluation of selected serum mineral concentrations and their association with insulin resistance in obese adolescents. Eur Rev Med Pharmacol Sci 17, 2396–2400. 24065235

[41] Laires MJ, Moreira H, Monteiro CP, (2004) Magnesium, insulin resistance and body composition in healthy postmenopausal women. J Am Coll Nutr 23, 510S–513S. 15466953 10.1080/07315724.2004.10719391

[42] Lu L, Chen C, Yang K, (2020) Magnesium intake is inversely associated with risk of obesity in a 30-year prospective follow-up study among American young adults. Eur J Nutr 59, 3745–3753. 32095867 10.1007/s00394-020-02206-3 PMC7483156

[43] Jiang S, Ma X, Li M, (2020) Association between dietary mineral nutrient intake, body mass index, and waist circumference in U.S. adults using quantile regression analysis NHANES 2007–2014. PeerJ 8, e9127. 32411541 10.7717/peerj.9127 PMC7204818

[44] Morais JBS, de Freitas TEC, Severo JS, (2019) No difference in magnesium intake between obese women and healthy controls. Int J Vitam Nutr Res 89, 118–124. 30982446 10.1024/0300-9831/a000413

[45] Vitale M, Masulli M, Cocozza S, (2016) Sex differences in food choices, adherence to dietary recommendations and plasma lipid profile in type 2 diabetes – the TOSCA.IT study. Nutr Metab Cardiovasc Dis 26, 879–885. 27212622 10.1016/j.numecd.2016.04.006

[46] Beardsworth A, Bryman A, Keil T, (2002) Women, men and food: the significance of gender for nutritional attitudes and choices. Br Food J 104, 470–491.

