# Supplementary material for: Relationship between overweight and obesity and insufficient micronutrient intake: a nationwide study in Taiwan
Source: J Nutr Sci. 2023 Apr 14;12:e48. doi: 10.1017/jns.2023.31 (PMC10131054; doi:10.1017/jns.2023.31)
Supplement: Supplementary file 1 [file S2048679023000319sup001.docx]

**Table S1. Taiwanese Dietary Reference Intakes of selected micronutrients**

| **Nutrients** | **Dietary Reference Intakes** | **Reference** |
| --- | --- | --- |
| Vitamin A | Aged 19 and above: 600 μg RE for men, 500 μg RE for women. | RDA |
| Vitamin C | Aged 19 and above: 100 mg. | RDA |
| Vitamin D | Aged 19 to 50: 10 μg;  Aged 51 and above: 15 μg. | AI |
| Vitamin E | Aged 19 and above: 12 mg α-TE. | AI |
| Vitamin B1 | Aged 19 and above: 1.2 mg for men, 0.9 mg for women. | RDA |
| Vitamin B2 | Aged 19 and above: 1.3 mg for men, 1.0 mg for women. | RDA |
| Vitamin B3 | Aged 19 and above: 16 mg NE for men, 14 mg NE for women. | RDA |
| Vitamin B6 | Aged 19 to 50: 1.5 mg;  Aged 51 and above: 1.6 mg. | RDA |
| Vitamin B12 | Aged 19 and above: 2.4 μg. | RDA |
| Iron | Aged 19 to 50: 10 mg for men, 15 mg for women;  Aged 51 and above: 10 mg. | RDA |
| Magnesium | Aged 19 to 50: 380 mg for men, 320 mg for women;  Aged 51 to 70: 360 mg for men, 310 mg for women;  Aged 71 and above: 350 mg for men, 300 mg for women. | RDA |
| Zinc | Aged 19 and above: 15 mg for men, 12 mg for women. | AI |
| Calcium | Aged 19 and above: 1000 mg. | AI |
| Phosphorus | Aged 19 and above: 800 mg. | AI |

RE: retinol equivalent; α-TE: α-tocopherol equivalent; NE: niacin equivalent; RDA: recommended dietary allowance; AI: adequate intakes.

**Table S2.** Micronutrient intakes of men and women in the current study.

|  | **Nutrient intakes** | |
| --- | --- | --- |
| **Nutrients** | **Men** | **Women** |
| Vitamin A | 632.17 (978.16) μg RE. | 592.57 (936.93) μg RE. |
| Vitamin C | 116.47 (144.43) mg. | 111.46 (157.54) mg. |
| Vitamin D | 3.76 (6.63) μg. | 3.17 (5.78) μg. |
| Vitamin E | 8.15 (6.04) mg α-TE. | 7.09 (5.62) mg α-TE. |
| Vitamin B1 | 1.39 (0.99) mg. | 1.06 (0.78) mg. |
| Vitamin B2 | 1.15 (0.89) mg. | 1.03 (0.75) mg. |
| Vitamin B3 | 17.82 (13.73) mg NE. | 13.86 (10.70) mg NE. |
| Vitamin B6 | 1.96 (1.32) mg. | 1.58 (1.08) mg. |
| Vitamin B12 | 3.39 (4.64) μg. | 2.68 (3.97) μg. |
| Iron | 14.56 (10.34) mg. | 12.04 (8.74) mg. |
| Magnesium | 305.01 (217.32) mg. | 255.15 (171.75) mg. |
| Zinc | 12.27 (7.47) mg. | 9.39 (5.45) mg. |
| Calcium | 467.51 (457.31) mg. | 435.92 (413.57) mg. |
| Phosphorus | 1202.76 (735.84) mg. | 957.02 (575.33) mg. |

RE: retinol equivalent; α-TE: α-tocopherol equivalent; NE: niacin equivalent.

The values were presented as median (interquartile range).

The Nutrition and Health Survey in Taiwan (NAHSIT) 2013-2016 included 11,072. participants aged 2month and above.

Participants aged 18 and below were excluded (*n*=5,302)

5,770 participants aged 19 and above were included.

Participants without 24-hour recall data were excluded (*n*=26).

5,744 participants with 24-hour dietary recall data.

Participants without physical examination data (body mass index or waist circumference) were excluded (*n*=2,669).

3,075 participants with complete data were finally included.

Figure S1. Diagram of the sample selection process for this study.

3,075　有BMI以及WC
